# The mediating effects of post-pandemic health promotion behaviors in the relationship between anxiety and quality of life in young adults in South Korea: a cross-sectional study

**DOI:** 10.7586/jkbns.24.006

**Published:** 2024-05-17

**Authors:** Hyang-Suk Choi, Myoung-Lyun Heo

**Affiliations:** 1 Department of Health Administration, Baekseok Culture University, Cheonan, Korea; 2 Department of Nursing, Jeonju University, Jeonju, Korea

**Keywords:** Young adult, Anxiety, Quality of life, Health promotion

## Abstract

**Purpose:**

This study aimed to investigate the mediating effects of health promotion behavior (HPB) in the relationship between anxiety and quality of life (QoL) in young adults living in the post-pandemic era.

**Methods:**

A cross-sectionaldescriptiveonlinesurveydesign was utilized. Data on anxiety, QoL, HPB, and demographic characteristics were collected from 213 adults aged 19–35 years in Korea via an online survey in January 2024. The collected data were analyzed using SPSS 27.0 and PROCESS MACRO 4.2 software.

**Results:**

Strong correlations were observed among anxiety, QoL, and post-pandemic HPB (PP-HPB) in young adults, andanxiety and PP-HPB were identified as significant predictors of QoL. The total effect of anxiety on QoL was significant (B = −1.40, bootstrapped SE = 0.10), with both the direct effect (B = −0.70, bootstrapped SE = 0.09) and the indirect effect (B = −0.70, bootstrapped SE = 0.11) being significant. This suggests that PP-HPB partially mediated the relationship between anxiety and QoL.

**Conclusion:**

This study highlights the importance of strengthening HPB with consideration of life changes since the coronavirus disease 2019 pandemic to improve QoL among young adults with anxiety.

## INTRODUCTION

Strict social distancing policies were enforced worldwide in the initial period of the coronavirus disease 2019 (COVID-19) pandemic [1]. These measures required individuals to minimize physical interactions and adopt an online-based lifestyle, and facilitated by rapid technological advancement and adaptation, this shift heralded the beginning of the post-pandemic era [2].

While the acceleration of technological progress has transformed academic and professional environments [2], offering benefits such as improved work–life balance and satisfaction owing to flexible work arrangements [3], issues such as lack of physical activity, job loss, and isolation have also led to negative health consequences, including depression and anxiety [4], directly impacting quality of life (QoL) [5]. QoL, defined as an individual’s perceived state of well-being [6], is crucial to health, as those with lower QoL tend to be more sensitive to stress and experience disruptions in daily living [7]. With the current climate of rapid technological evolution, competition, and uncertainty, the absence of individual or policy-driven efforts could further deteriorate QoL [8].

The young adult period—approximately from age 20 to the early-to-mid-30s—is marked by significant life transitions, such as independence from parents, higher education enrollment and graduation, employment, and marriage [9]. Individuals of this age, which includes students, job seekers, and the employed, face heightened anxiety from a combination of socio-structural issues, such as more tenuous employment conditions and safety [10]. Moreover, the COVID-19 pandemic has exacerbated health-related anxiety [11,12] and increased the reliance on mobile devices among young adults raised in a digital culture. Coupled with reduced physical activity and opportunities for direct contact with others, this reliance on mobile devices has led to increased mental health issues, including loneliness and anxiety [13].

There is a lack of research on young adults’ QoL, largely owing to the socially constructed image that young adults have high self-rated health and are healthy. However, recent studies have indicated a rise in the prevalence of chronic diseases such as hypertension and diabetes mellitus among young adults, suggesting that both their physical and mental health, and consequently their QoL, are at risk [14]. The World Health Organization is actively working to reduce health disparities, as health is one of the most fundamental human rights. Interventions that address health disparities ultimately boost global productivity and economic benefits [15]. Thus, from the perspective of addressing health disparities, there is a compelling need to prioritize the health and QoL of young adults, who, despitetheir pivotal role as the forthcoming generation, have been relatively overlooked.

Pender et al. (2006) [16] conceptualized health promotion as optimizing health and creating healthy environments, emphasizing the importance of behavioral change for health promotion. According to previous studies, anxiety has been identified as a predictor of health-promoting behaviors [17,18], and it has been established that health-promoting behaviors are significant predictors of QoL [19,20]. Therefore, based on this evidence, we can hypothesize a mediating effect of health-promoting behaviors in the relationship between anxiety and QoL. Particularly, health promotion behaviors (HPB) are a modifiable factor that can be influenced by health education and systematic management and support [21]. Thus, shedding light on HPB’ role in the relationship between anxiety and QoL is crucial to devising strategies to enhance QoL in a post-pandemic society characterized by heightened anxiety. As previously mentioned, following the pandemic, numerous changes have emerged, with young adults adapting more quickly to these changes at the forefront compared to other generations. Therefore, this study thus aimed to explore the role of post-pandemic HPB (PP-HPB), a concept that expands on the traditional concept of HPB, in young adults using a new instrument that focuses on HPB related to mobile device use. Accordingly, the following research question was developed: Do PP-HPB have a mediating effect on the relationship between anxiety and QoL in the digitally native young adult population?

## METHODS

### 1. Study design

**Figure 1.**
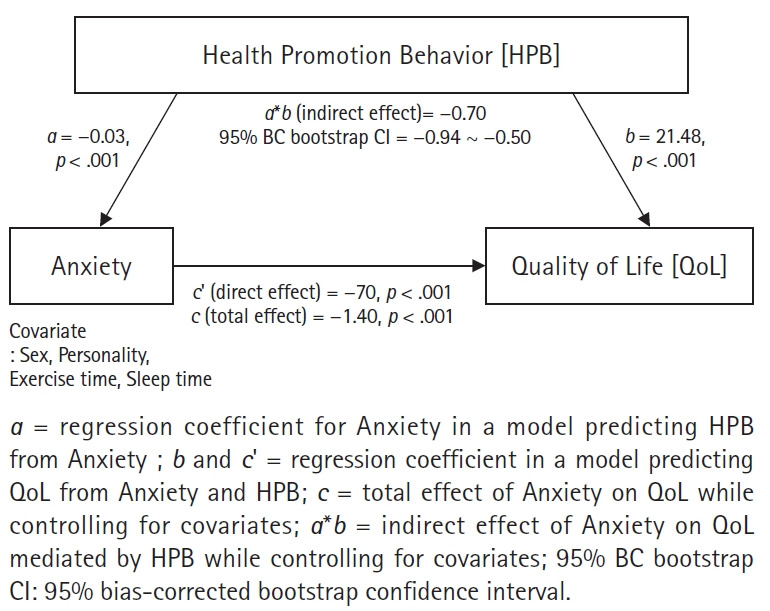
Model examining the mediating effect of health promotion behaviors (HPB) in the relationship between anxiety and quality of life (QoL)

This study applied a cross-sectional descriptive study design using an online survey to investigate the mediating effects of PP-HPB in the relationship between anxiety and QoL in young adults.The study’s conceptual framework is illustrated in Figure 1.

### 2. Study participants and data collection

Adults aged 19–35 years residing in Korea who provided informed consent to participate in the study after viewing an online recruitment announcement were enrolled. Andto verify compliance with the selection criteria, two separate questions about age were inserted at the beginning and end of the survey in different formats. The sample size was determined using the G*Power 3.1.9.7 program. For a multiple linear regression with an effect size of .15, power of .95, and predictive variables 14, the minimum sample size was calculated as 194. Considering a 10% dropout rate, the target sample size was set at 213. The study procedure and methods were approved by the Institutional Review Board (IRB) of the author’s institution before recruiting participants. An information sheet explaining the collection of personal information, anticipated risks or side effects, and freedom to withdraw from the study was also provided to potential participants, and those who consented to these measures proceeded with the online survey. The survey was conducted on January 8, 2024, and the survey writing took approximately 20 minutes. All participants were given a coffee voucher. None of the collected surveys had careless responses, so all 213 questionnaires were included in the final analysis.

### 3. Instruments

#### 1) Anxiety

The Koreanversionof the Self-rating Anxiety Scale (K-SAS) constitutes a version of the instrument developed by Zung [22] validated in the Korean context [23]. This tool encompasses all symptoms of general anxiety and contains 20 items. Each item is rated on a four-point Likert scale, consisting of 1-“never,” 2-“sometimes,” 3-“frequently,” and 4-“always.” A higher score indicates greater levels of anxiety. The Cronbach’s ⍺ was .98 in Lee (1995) [23] and .92 in this study.

#### 2) QoL

The Korean version of the QoL Survey (K-QoLS) is a version of the tool developed by Gill et al. [24] validated in the Korean context [25]. This 32-item tool contains four items for integrated QoL, five for social QoL, five for spiritual QoL, five for emotional QoL, five for cognitive QoL, five for physical QoL, and seven for activities of daily living (ADL). Each item is rated on a five-point Likert scale: “low,” “below average,” “average,” “above average,” and “very high.” A higher score indicates higher QoL. The Cronbach’s ⍺ was .98 in Park et al. (2015) [25] and .97 in this study.

#### 3) PP-HPB

PP-HPB were assessed using a new tool developed by Heo et al [26] for young adults who experienced the pandemic. This six-factor, 27-item tool consists of eight items for psychosocial health, five for personal hygiene, five for dietary habits, two for health management, four forusingmobiledevices, and three forphysicalactivity. Each item is rated on a five-point Likert scale ranging from 1-“strongly disagree” to 5-“strongly agree.” A higher score indicates greater levels of HPB. The Cronbach's ⍺ was .90 at the time of development and .94 in this study.

### 4. Data analysis

The collected data were analyzed using IBM SPSS 27.0 and PROCESS MACRO 4.2. Participants’ general characteristics and K-SAS, K-QoLS, and PP-HPB scores were evaluated using frequency analysis and descriptive statistics. Differences in K-SAS, K-QoLS, and PP-HPB scores according to general characteristics were analyzed with the independent *t*-test and one-way analysis of variance. The correlations among the variables were analyzed using Pearson correlation coefficient. The variance inflation factor (VIF) and autocorrelation (Durbin-Watson) were checked before analyzing the mediating effects, and the mediating effects were analyzed using PROCESS MACRO model 4. Statistical significance of the indirect effect was tested with bootstrapping using 5,000 samples at a 95% confidence interval (CI). The statistical significance levels set at .05 were two-sided.

### 5. Ethical considerations

This study was conducted after obtaining approval from the IRB of the university to which the researchers are affiliated (IRB NO. jjIRB-231214-HR-2023-1112). The subjects were provided with documents in the form of postings on an online bulletin board, explaining the purpose and procedures of the research as well as the possibility of withdrawal. Surveys were conducted only if the participants consented voluntarily, and participants were provided with coffee coupons as a token of appreciation for their participation in the research.

## RESULTS

### 1. Participants’ general characteristics

Table 1 provides the general characteristics of the 213 participants. The mean age was 28.17 ± 3.23 years, with 15.0% aged under 25, 40.9% aged 25-30, and 44.1% aged 31-35. There were 47.4% male and 52.6% female participants, with 17.4% being unemployed or students, and 82.6% employed. Education levels included 10.8% with a high school diploma or lower, 11.7% current college students, and 77.5% with a college degree or higher. 56.8% of participants lived alone, while the remainder lived with family, friends, or coworkers. The average sitting time was 7.03 ± 2.85 hours, with 45.1% sitting for more than 7 hours. Monthly income levels were under 500,000 KRW for 8.0%, 500,000-2,000,000 KRW for 13.2%, and over 2,000,000 KRW for 78.9%. Regarding personality type, 66.2% identified as introverted. The average daily usage of electronic devices was 6.11 ± 3.35 hours, with 37.1% using devices for over 6 hours. The mean daily exercise duration was 1.03 ± 0.61 hours, with 83.1% exercising for an hour or less, while the mean daily sleep duration was 7.21 ± 1.00 hours, with 68.1% sleeping for 7 hours or less.

### 2. Degree of anxiety, QoL, and HPB

Regarding levels of anxiety, QoL, and HPB, anxiety and OoL were analyzed using total scores, while the average score was used for HPB according to the method employed by the developers (Table 2). The mean anxiety score was 36.38 ± 10.28, and the mean QoL score was 112.39 ± 21.53. The mean scores for each domain of QoL were as follows: 17.27 ± 3.55 for physical, 17.83 ± 3.62 for social, 17.46 ± 3.80 for emotional, 17.90 ± 3.36 for cognitive, 16.70 ± 4.15 for mental, 10.67 ± 2.42 for ADL, and 14.56 ± 3.10 for global QoL. The mean HPB score was 3.59 ± 0.60, with 3.75 ± 0.63 for emotional and social health, 3.82 ± 0.73 for personal hygiene, 3.31 ± 0.86 for dietary habits, 3.54 ± 0.83 for health management, 3.57 ± 0.76 for using mobile devices, and 3.31 ± 0.84 for physical activity. The score was the highest for personal hygiene and the lowest for dietary habits and physical activity.

### 3. Differences in anxiety, QoL, and HPB according to general characteristics

Table 1 shows the differences in anxiety, QoL, and HPB according to participants’ general characteristics. Anxiety significantly differed according to sex (*t* = 2.98, *p* = .003), personality (*t* = 3.56, *p* < .001), exercise time (*t* = 6.12, *p* < .001), and sleep time (*t* = 3.09, *p* = .002). QoL significantly differed according to sex (*t* = −3.59, *p* < .001), personality (*t* = −3.76, *p* < .001), exercise time (*t* = −6.96, *p* < .001), and sleep time (*t* = −4.98, *p* < .001). HPB also significantly differed according to sex (*t* = −5.15, *p* < .001), personality (*t* = −3.29, *p* < .001), exercise time (*t* = −5.21, *p* < .001), and sleep time (*t* = −4.10, *p* < .001).

### 4. Correlations among anxiety, QoL, and HPB

Table 2 shows the correlations among scores for anxiety, QoL, and HPB. Anxiety was significantly negatively correlated with QoL (*r* = −.77, *p* < .001) and HPB (*r* = −.68, *p* < .001), while HPB was significantly positively correlated with QoL (*r* = −.85, *p* < .001).

### 5. Mediating effects of HPB in the relationship between anxiety and QoL

Multicollinearity was tested before conducting the mediation analysis. Tolerance was .53, and VIF was smaller than 10, at 1.87. The Durbin-Watson statistic was close to 2, at 2.281, confirming the normality and equal variance of the residuals. Per the Hayes [27] protocol, mediation analysis was performed using Process Macro model 4 with bootstrapping at a sample size of 5,000. The general characteristics that significantly differed in relation to HPB, namely sex, personality, exercise duration, and sleep duration, were dummy-coded. The stepwise analysis results are shown in Table 3.

Model 1 captured the effects of anxiety on QoL after controlling for participants’ general characteristics; anxiety had a significant effect (B = −1.40, SE = 0.10, *p* < .001). Model 2 captured the effects of anxiety on HPB after controlling for general characteristics; a significant effect (B = −0.03, SE = 0.10, *p* < .001) was found for anxiety. Model 3 captured the effects of anxiety and HPB on QoL after controlling for general characteristics; both anxiety (B = −0.70, SE = 0.09, *p* < .001) and HPB had a significant effect (B = 21.48, SE = 1.65, *p* < .001), suggesting that anxiety and HPB are significant predictors of QoL.

Finally, the significance of effect decomposition and mediating effects was tested (Table 4). The total effect of anxiety on QoL was B = −1.40, Boot SE = 0.10, and was significant, as the CI did not include 0. The direct effect was B = −0.70, Boot SE = 0.09, and was significant, as the CI did not include 0. The indirect effect was B = −0.70, Boot SE = 0.11, and was significant, as the CI did not include 0. Thus, HPB was found to have a partial mediating effect.

## DISCUSSION

This study investigated the mediating effects of HPB in the relationship between anxiety and QoL in young adults. The key findings are discussed below.

Young adulthood is characterized by a marked increase in anxiety levels [28], and elevated anxiety is linked to diminished social and psychological QoL [29,30]. This study also found that anxiety was significantly associated with QoL among young adults, suggesting the need for strategies to alleviate anxiety to increase QoL. However, the roots of anxiety in young adults range from solvable personal issues to intractable societal challenges such as job scarcity, competition, and financial insecurity [10]; therefore, directly lowering anxiety by eliminating its causes is difficult.

In our study, the K-QoLS score significantly increased when the impact of K-SAS was mediated by PP-HPB, suggesting that PP-HPB has a partial mediating effect and that HPB can indirectly mitigate the adverse effects of anxiety. Thus, encouraging such behaviors among anxious young adults is a pivotal strategy for enhancing QoL. Particularly, the PP-HPB instrument used in this study reflects the online-centric lifestyle and health perceptions of the younger generation [26], providing a rationale for actively offering HPB intervention programs to young adults in today’s rapid-paced society.In other words, while anxiety and QoL are typically addressed as psychological variables, based on the findings of this study, managing anxiety and QoL in young adults requires not only psychological approaches but also strengthening health promotion strategies that encompass various factors such as dietary habits, physical activity, and more.

However, caution is advised in interpreting the study’s results. Namely, the correlation between the HPB and QoLS was high. A review of the survey items indicated that the correlation coefficient between the two instruments was above .80, although the two measured different concepts. Thus, multicollinearity was separately verified via the tolerance, VIF, and Durbin-Watson values using regression analysis on SPSS. Although we confirmed the absence of multicollinearity, the correlation between the two factors should be carefully considered in future studies that investigate these concepts.

In the wake of COVID-19, rapid societal shifts have altered vocational structures and personal values, exacerbating the uncertainty and anxiety experienced by young adults. The mean K-SAS score among our participants was 36.38. Considering that a cut-off score of 36 is used for diagnosing anxiety and that the control group with no mental disorders had a mean score of 33 at the time of the tool’s development [22,31], the finding that the average score exceeded the diagnostic threshold can indicate elevated anxiety levels. Although there is debate on raising the cut-off score [32], the importance of this study lies in its documentation of anxiety levels among young adults, a population for which SAS scores are seldom reported.

In addition, we found the mean K-QoLS score to be 112.39. Research in nursing or public health often focuses on measuring health-related QoL, the K-QoLS encompasses a broad spectrum of aspects, including general, social, psychological, emotional, cognitive, and physical health, as well as ADLs. Given the absence of similar research targeting young adults, the scores could not be compared directly. Nevertheless, it should be noted that because QoL encompasses various domains, focusing solely on physical health can be limiting [33]. Furthermore, some argue that perceived physical health is not the most crucial aspect of QoL [34], indicating the need for broader conceptual measurements and analyses in assessing QoL among young adults.

HPB has been found to have a positive impact on QoL [35]. While the current study found a strong correlation between HPB and QoL, one notable difference from previous studies is that we analyzed PP-HPB scores, which were developed specifically for young adults, and incorporated questions related to health behaviors using mobile devices. The analysis indicated that dietary habits and physical activity levels were the lowest of all the categories, consistent with findings from similar studies [26,36]. These similar results across multiple studies highlight the challenges to basic health behaviors such as diet and physical activity due to the prevalence of online-centric lifestyles and convenience foods [36], suggesting that improvements in these areas could potentially influence the relationship between anxiety and QoL.

Participant characteristics, namely, sex, personality, exercise duration, and sleep duration, showed significant differences across all three concepts of anxiety, QoL, and PP-HPB. While these variables were controlled for in the mediation analysis, sleep remained significant in the pathway analysis examining the effects of anxiety and PP-HPB on QoL. Thus, sleep should be considered when developing interventions or in subsequent research. The US Centers for Disease Control and Prevention [37] recommends adults aged 18–60 get at least 7 hours of sleep, yet in our study, 68.1% of participants reported sleeping less than seven hours. Excessive smartphone use has been directly linked to insomnia [38], and with smartphones becoming integral to all aspects of daily life post-pandemic [2], interventions to improve HPB should incorporate strategies to promote positive behaviors related to smartphone use and sleep.

In terms of study limitations, the study population consisted of a subset of young adults in Korea, which limits the generalizability of the findings. Further, the results should be interpreted with caution, considering that a high percentage of our participants were employed, despite the diverse nature of the young adult population that encompasses students, workers, and job seekers. However, this study makes a key contribution by providing a baseline for subsequent studies attempting to shed light on the importance of health management for enhancing young adults’ QoL worldwide based on data from this highly digitalized country.

## CONCLUSION

This study found that anxiety and HPB influenced QoL in young adults and that HPB mediated the relationship between anxiety and QoL. These results underscore the need for strategies to enhance HPB in consideration of the post-pandemic lifestyle to enhance QoL among young adults with anxiety that cannot be addressed in the short term. Considering the limited sample of Korean adults, future studies should include different ethnicities and countries. We hope our findings serve as grounds for research aimed at developing programs to enhance HPB among young adults.

## Figures and Tables

**Table 1.** Anxiety, QoL, and HPB Scores according to Participants’ General Characteristics (N = 213)

Characteristics	Categories	n (%)	Anxiety (K-SAS)		QoL (K-QoLS)		HPB (PP-HPB)	
			M ± SD	t (*p)*	M ± SD	t (*p)*	M ± SD	t (*p)*
Age (yr) (M ± SD; 28.17 ± 3.23)	< 25	32 (15.0)	34.00 ± 10.27	1.45 (.238)	119.03 ± 16.90	1.90 (.151)	3.66 ± 0.52	0.28 (.759)
	25-30	87 (40.9)	37.54 ± 11.39		110.48 ± 23.39		3.56 ± 0.64	
	31-35	94 (44.1)	36.13 ± 9.07		111.89 ± 20.90		3.59 ± 0.58	
Sex	Male	101 (47.4)	38.55 ± 9.43	2.98 (.003)	107.02 ± 18.46	-3.59 (< .001)	3.38 ± 0.51	-5.15 (< .001)
	Female	112 (52.6)	34.43 ± 10.65		117.23 ± 22.98		3.78 ± 0.61	
Occupation	No	37 (17.4)	34.51 ± 9.60	-1.22 (.224)	113.92 ± 20.43	0.47 (.636)	3.54 ± 0.65	-0.57 (.569)
	Yes	176 (82.6)	36.78 ± 10.40		112.07 ± 21.80		3.60 ± 0.59	
Education level	≤ High school graduate	23 (10.8)	41.09 ± 12.66	2.98 (.053)	106.13 ± 21.16	1.82 (.164)	3.40 ± 0.51	1.37 (.255)
	College student	25 (11.7)	34.52 ± 10.31		117.96 ± 16.95		3.64 ± 0.56	
	> College graduate	165 (77.5)	36.01 ± 9.78		112.42 ± 22.07		3.61 ± 0.61	
Household composition	Alone	121 (56.8)	37.00 ± 10.21	1.00 (.318)	111.98 ± 21.57	-0.32 (.753)	3.57 ± 0.62	-0.53 (.599)
	Family, friend, colleague	92 (43.2)	35.58 ± 10.36		112.92 ± 21.58		3.61 ± 0.57	
Sitting time/day (hr) (M ± SD; 7.03 ± 2.85)	≤ 7	117 (54.9)	37.54 ± 9.93	1.82 (.070)	110.50 ± 20.50	-1.42 (.157)	3.52 ± 0.54	-1.70 (.090)
	> 7	96 (45.1)	34.98 ± 10.57		114.70 ± 22.61		3.67 ± 0.65	
Activities primarily done while sitting	Studying (work)/hobbies	183 (85.9)	36.55 ± 10.15	0.57 (.572)	112.84 ± 21.12	0.76 (.451)	3.61 ± 0.59	1.05 (.295)
	Using electronic devices	30 (14.1)	35.40 ± 11.14		109.63 ± 24.10		3.48 ± 0.66	
Monthly income (won)	≤ 500,000	17 (8.0)	36.53 ± 10.78	0.50 (.607)	109.71 ± 21.12	0.53 (.587)	3.45 ± 0.67	1.09 (.338)
	500,000-2,000,000	28 (13.1)	34.57 ± 10.89		109.29 ± 25.63		3.49 ± 0.61	
	> 2,000,000	168 (78.9)	36.67 ± 10.15		113.18 ± 20.89		3.62 ± 0.58	
Personality	Introverted	141 (66.2)	38.13 ± 10.07	3.56 (< .001)	108.54 ± 20.96	-3.76 (< .001)	3.49 ± 0.57	-3.29 (.001)
	Extroverted	72 (33.8)	32.97 ± 9.87		119.93 ± 20.75		3.77 ± 0.60	
Electronic device usage time/day (hr) (M ± SD; 6.11 ± 3.35)	≤ 6	134 (62.9)	36.79 ± 9.56	0.72 (.475)	111.21 ± 19.10	-0.97 (.333)	3.54 ± 0.51	-1.31 (.191)
	> 6	79 (37.1)	35.70 ± 11.42		114.39 ± 25.13		3.66 ± 0.72	
Exercise time/day (hr) (M ± SD; 1.03 ± 0.61)	≤ 1	177 (83.1)	37.79 ± 10.32	6.12 (< .001)	108.85 ± 20.89	-6.96 (< .001)	3.50 ± 0.57	-5.21 (< .001)
	> 1	36 (16.9)	29.47 ± 6.70		129.81 ± 15.40		4.03 ± 0.53	
Sleep time/day (hr) (M ± SD; 7.21 ± 1.00)	≤ 7	145 (68.1)	37.81 ± 10.34	3.09 (.002)	107.62 ± 21.54	-4.98 (< .001)	3.48 ± 0.60	-4.10 (< .001)
	> 7	68 (31.9)	33.35 ± 9.53		122.56 ± 17.76		3.82 ± 0.52	

QoL= Quality of life; HPB = Health promotion behavior; K-SAS = Korean version of the self-rating anxiety scale; K-QoLS = Korean version of the quality of life survey; PP-HPB = Post-pandemic HPB; M = Mean; SD = Standard deviation.

**Table 2.** Levels and Correlations of Anxiety, QoL, and HPB in Participants (N = 213)

Variables	Min	Max	M ± SD	r (*p*)	
				Anxiety	QoL
Anxiety	20.00	68.00	36.38 ± 10.28	1	
QoL	39.00	153.00	112.39 ± 21.53	-.77 (< .001)	1
Physical	8.00	25.00	17.27 ± 3.55		
Social	6.00	25.00	17.83 ± 3.62		
Emotional	5.00	24.00	17.46 ± 3.80		
Cognitive	6.00	24.00	17.90 ± 3.36		
Spiritual	5.00	25.00	16.70 ± 4.15		
Activities of daily living	4.00	15.00	10.67 ± 2.42		
Integrated	4.00	20.00	14.56 ± 3.10		
HPB	2.04	4.78	3.59 ± 0.60	-.68 (< .001)	.85 (< .001)
Psychosocial health	2.25	5.00	3.75 ± 0.63		
Personal hygiene	2.00	5.00	3.82 ± 0.73		
Dietary habits	1.20	4.80	3.31 ± 0.86		
Health management	1.00	5.00	3.54 ± 0.83		
Using mobile devices	1.50	5.00	3.57 ± 0.76		
Physical activity	1.00	5.00	3.31 ± 0.84		

QoL = Quality of life; HPB = Health promotion behavior; Min = Minimum; Max = Maximum; M = Mean; SD = Standard deviation.

**Table 3.** Step-by-step Verification of the Mediation Effects Analysis (N = 213)

Model	B	SE	t	*p*	LLCI	ULCI	R^2^	F (*p*)
Model 1 (X: Anxiety -> Y: QoL)								
(Constant)	157.18	4.34	36.26	< .001	148.63	165.72	0.64	73.85 (< .001)
Anxiety	-1.40	0.10	-14.46	< .001	-1.60	-1.21		
Covariates								
Sex (ref. Male)	3.91	1.84	2.13	.034	0.29	7.53		
Personality (ref. Introverted)	3.09	1.97	1.57	.119	-0.80	6.98		
Exercise time (ref. ≤ 1 hour)	6.90	2.63	2.63	.009	1.73	12.07		
Sleep time (ref. ≤ 7 hours)	6.23	2.05	3.04	.003	2.18	10.28		
Model 2 (X: Anxiety-> Y: HPB)								
(Constant)	4.55	0.14	33.52	< .001	4.28	4.82	0.54	48.65 (< .001)
Anxiety	-0.03	0.00	-10.79	< .001	-0.04	-0.03		
Covariates								
Sex (ref. Male)	0.25	0.06	4.33	< .001	0.14	0.36		
Personality (ref. Introverted)	0.08	0.06	1.32	.190	-0.04	0.20		
Exercise time (ref. ≤ 1 hour)	0.22	0.08	2.65	.009	0.06	0.38		
Sleep time (ref. ≤ 7 hours)	0.12	0.06	1.81	.071	-0.01	0.24		
Model 3 (X: Anxiety, HPB-> Y: QoL)								
(Constant)	59.51	8.16	7.29	< .001	43.43	75.60	0.80	139.97 (< .001)
Anxiety	-0.70	0.09	-7.78	< .001	-0.88	-0.52		
HPB	21.48	1.65	13.03	< .001	18.22	24.73		
Covariates								
Sex (ref. Male)	-1.43	1.42	-1.01	.315	-4.24	1.37		
Personality (ref. Introverted)	1.35	1.47	0.92	.361	-1.55	4.25		
Exercise time (ref. ≤ 1 hour)	2.23	1.98	1.12	.263	-1.68	6.13		
Sleep time (ref. ≤ 7 hours)	3.73	1.54	2.43	.016	0.70	6.76		

QoL= Quality of life; HPB = Health promotion behavior; ref. = Reference; LLCI = Lower limit of confidence interval; ULCI = Upper limit of confidence interval.

**Table 4.** Significance of Effect Decomposition and Mediating Effects (N = 213)

Effect	Path	Effect	Boot SE	95% CI	
				Boot LLCI	Boot ULCI
Total effect	Anxiety→QoL	-1.40	0.10	-1.59	-1.21
Direct effect	Anxiety→QoL	-0.70	0.09	-0.88	-0.52
Indirect effects	Anxiety→HPB→QoL	-0.70	0.11	-0.94	-0.50

QoL = Quality of life; HPB = Health promotion behavior; Boot = Bootstrap; SE = Standard error; CI = Confidence interval; LLCI = Lower limit of confidence interval; ULCI = Upper limit of confidence interval.

## Data Availability

Please contact the corresponding author for data availability.
